# Proton pump inhibitors and histamine-2-receptor antagonists and pancreatic cancer risk: a nested case–control study

**DOI:** 10.1038/bjc.2011.511

**Published:** 2011-11-22

**Authors:** M C Bradley, L J Murray, M M Cantwell, C M Hughes

**Affiliations:** 1 Clinical and Practice Research Group, School of Pharmacy, Queen's University Belfast, 97 Lisburn Road, Belfast, Northern Ireland BT9 7BL, UK; 2 Cancer Epidemiology and Health Services Research Group, Centre for Public Health, Queen's University Belfast, Institute for Clinical Sciences, Royal Victoria Hospital, Belfast, Northern Ireland, BT12 6BJ, UK

**Keywords:** pancreatic cancer, proton pump inhibitors, histamine-2-receptor antagonists, GPRD, hypergastrinaemia

## Abstract

**Background:**

The relationship between use of proton pump inhibitors (PPIs) and histamine-2-receptor antagonists (H_2_RAs) and pancreatic cancer risk has yet to be examined. Data from a range of studies suggest biologically plausible mechanisms, whereby these drugs (or the conditions for which they are prescribed) may affect pancreatic cancer risk. The objective of this study was to investigate the relationship between use of PPIs/H_2_RAs and pancreatic cancer risk.

**Methods:**

A nested case–control study was conducted within the UK general practice research database (GPRD). Cases had a diagnosis of exocrine pancreatic cancer and controls were matched to cases on general practice site, sex and year of birth. Exposure to PPIs and to H_2_RAs since entry into GPRD until 2 years before the diagnosis date (corresponding date in controls) and in the 5 years before the diagnosis date were separately assessed. Conditional logistic regression analyses were used to generate odds ratios (ORs) and 95% confidence intervals (CIs) associated with PPI or H_2_RA use compared with nonuse.

**Results:**

Ever use of PPIs since entry into the GPRD (excluding the 2 years prior to diagnosis) was not associated with risk of pancreatic cancer; OR (95% CI) 1.02 (0.85–1.22). Neither the dose nor the duration of PPI or H_2_RA use was associated with pancreatic cancer risk. No consistent patterns of association were seen when cumulative exposure (dose and duration) to these drugs was examined separately or together.

**Conclusion:**

PPI/H_2_RA use, in a UK population, was not associated with pancreatic cancer risk.

Pancreatic cancer is rapidly fatal and is the fifth leading cause of cancer mortality in the western world. Almost 50% of patients present with distant metastases and little progress has been made in recent decades in the diagnosis or treatment of this cancer, resulting in poor survival rates (Jemal *et al*, 2007).

Proton pump inhibitors (PPIs) are some of the most commonly prescribed medications in North America (IMS Health, 2008) and the United Kingdom (National Health Service Business Services Authority, 2009). They are used in the treatment of gastrointestinal disorders, such as gastro-oesophageal reflux disease (DeVault and Castell, 2005; BMJ group, 2009). PPIs are potent suppressors of gastric acid and recently they have been used with increased frequency and for prolonged durations (Naunton *et al*, 2000). The long-term gastric hypoacidity associated with PPI use results in raised serum gastrin levels (hypergastrinaemia) (Klinkenberg-Knol *et al*, 1994). Histamine-2-receptor antagonists (H_2_RAs) are also used for similar indications, have been in use since the 1970s, and may induce hypergastrinaemia and hypoacidity (Wynn *et al*, 2007).

There are a number of potential mechanisms, whereby use of PPIs/H_2_RAs may influence pancreatic cancer risk (Risch, 2003). Gastric hypoacidity favours the overgrowth of bacteria in the stomach (Fried *et al*, 1994; Thorens *et al*, 1996), which results in the production of N-nitrosamines (Yeomans *et al*, 1995), although there is some evidence to the contrary (Vermeer *et al*, 2001). Pancreatic ductal adenocarcinomas have been seen to develop in mice and human pancreatic cells exposed to nitrosamines (Parsa *et al*, 1981; Parsa, 1987). PPI-/H_2_RA-related hypergastrinaemia may also contribute to pancreatic cancer carcinogenesis. Gastrin appears to have a role in the development and progression of gastrointestinal malignancies (Chao and Hellmich, 2010). Gastrin receptors are expressed in human pancreatic cancer cells (Smith *et al*, 1994), and gastrin has been seen to stimulate the growth of human pancreatic cancer cells in culture and in tumours transplanted into nude mice (Smith *et al*, 1995). These effects were blocked by gastrin-receptor antagonists (Smith *et al*, 1995), such as gastrazole (Roberts *et al*, 2002), which in a small randomised controlled trial increased the survival time among pancreatic cancer patients (Chau *et al*, 2006) Other studies refute this theory (Chu *et al*, 1997).

PPI/H_2_RA use increases secretin levels, which may influence pancreatic cell growth (Sarfati *et al*, 1985; Howatson and Carter, 1987).

The relationship between use of PPI/H_2_RAs and pancreatic cancer risk has yet to be examined, despite widespread use of these drugs and data from a range of studies suggesting biologically plausible mechanisms, whereby these drugs (or the conditions for which they are prescribed) may affect pancreatic cancer risk. We examined this relationship in a large nested case–control study within the UK general practice research database (GPRD).

## Materials and methods

We undertook a nested case–control study within the UK GPRD. The GPRD collects data from around 500 general practices in the United Kingdom, covers about 8% of the population and is broadly representative of the UK population. The data obtained are assessed in terms of completeness, continuity and plausibility, and general practices that meet predefined standards are registered as ‘up to standard’ (UTS) practices. The information recorded in GPRD includes demographic information, clinical diagnoses, referral information, specialty consultation notes, results of laboratory tests and hospital discharge information. The Read and Oxford Medical Information System codes are used to classify medical diagnoses. The GPRD also contains details of all prescriptions issued for patients registered with the participating practices (García Rodríguez and Pérez Gutthann, 1998). The high quality of GPRD prescription and diagnosis information has been documented (Jick *et al*, 1991, 1992). Ethical approval for all observational research using GPRD data has been obtained from a multicentre research ethics committee.

### Study population

The study included patients from UTS practices with diagnostic codes for primary malignant neoplasia of the exocrine pancreas, with a date of diagnosis/index date (first recorded occurrence of a pancreatic cancer code) between January 1995 and June 2006. Data were extracted from the GPRD in February 2007. Patients aged 85 or older were excluded as were cases without at least 5 years of UTS data collection prior to the index date. Controls with no GPRD record of pancreatic cancer were selected and matched to cases on year of birth, sex and general practice site. Controls also had at least 5 years of UTS data collection before their index date (date of pancreatic cancer diagnosis in their matched case). Up to seven controls were matched for each case.

### Review of cancer codes

Pancreatic cancer is difficult to conclusively diagnose without histological or cytological examination of relevant specimens and these specimens are often not available. Pancreatic cancer may therefore be confused with primary malignancies in anatomically related organs or with secondary tumours. In one UK cancer registry (unrelated to this study), only 57% of the patients had histological or cytological confirmation of their diagnosis (Kinnear *et al*, 2005). Data on histological or cytological confirmation of pancreatic cancer were not available from GPRD, at the time the data were obtained, as there was no routine linkage to cancer registries. In order to minimise misclassification of pancreatic cancer within this study, all cancer codes recorded in the medical records of potential cases were reviewed. Only cases with consistently recorded pancreatic cancer codes were included. All other cases, for example, patients with codes for both pancreatic cancer and cholangiocarcinoma, or carcinoma of bile duct, gallbladder and so on, and their matched controls were excluded from the dataset. Periampullary tumours were also excluded. Cases and controls with a prior history of cancer (at least one year before the index date) were also identified by review of cancer codes. These patients were flagged as having a previous cancer but were not excluded from the dataset.

### Exposure to PPIs/H_2_RAs

The primary exposures of interest were PPI and H_2_RA use since entry into the database until 2 years before the index date. PPIs/H_2_RAs were defined as listed in the British National Formulary 2009 (British Medical association and Royal Pharmaceutical Society of Great Britain, 2009), a national reference source for information on drugs. *A priori*, we decided that a positive association between pancreatic cancer risk and use of antisecretory drugs, in the 2 years preceding the index date, may be observed as pancreatic cancer symptoms often present as non-specific abdominal symptoms and antisecretory drugs may have been used to alleviate these symptoms. As a result, PPI/H_2_RA use in the 2 years before diagnosis was excluded from the analyses. PPI/H_2_RA use individually in the 5 years before the index date (excluding the 2 years before) was also examined, as well as combined dose and duration of PPI/H_2_RA use since entry into the database. The total exposure (dose and duration) to antisecretory drugs (PPIs and H_2_RAs) was also examined. The drug history for subjects in the study population was reviewed and data were extracted on all PPIs/H_2_RAs prescribed for cases and controls. PPI/H_2_RA use was expressed in units of defined daily dose (DDD), a validated measure of drug consumption maintained by the World Health Organisation. It is the assumed average maintenance dose per day of a drug that is used for its main indication in adults (World Health Organisation Working Group, 2008), as the DDD of a drug may be assumed to be functionally equivalent to the DDD of any other drug used for a similar purpose.

The number of DDDs for each PPI/H_2_RA prescription issued was calculated by multiplying the dose of the PPIs/H_2_RAs prescribed by the quantity given and dividing this by the DDD value assigned to that drug. For each time period under study, the total number of DDDs, for each PPI/H_2_RA prescribed, and for all PPIs/H_2_RAs combined, were calculated for each case and control. Total dose of PPIs/H_2_RAs (expressed in DDDs), in the periods under study, were categorized into approximate quartiles on the basis of total PPI/H_2_RA use within the controls. Cumulative duration of any PPI/H_2_RA use was calculated as the total number of intended treatment days. Total duration of any PPI/H_2_RA use, in the periods under study, was also categorized into approximate quartiles, on the basis of duration of treatment within the controls.

Exposure to PPIs/H_2_RAs at any time prior to the index date excluding the 2 years before the index date was further categorized according to both dose and duration of use. The median number of DDDs, per day, of PPI/H_2_RA exposure in the controls during this period was 1 DDD per day, therefore low dose PPI/H_2_RA exposure was classified as the use of <1.0 DDD of PPIs/H_2_RAs per day and high dose as >1.0 DDDs per day. Categories for duration of low and high PPIs/H_2_RAs use were subsequently created; no use or use for <1 year, use for 1–3 years, use for 3–5 years and use for ⩾5 or more years (Yang *et al*, 2007). The total use of PPIs and H_2_RAs was calculated by summing the total number of DDDs/days of use since entry into the database.

### Statistical analysis

Conditional logistic regression was used to calculate odds ratios (ORs) and 95% confidence intervals (CIs) for the associations between disease status and use of PPIs/H_2_RAs. Models were constructed for ever/never use of all PPIs/H_2_RAs separately. Dose and duration of PPI/H_2_RA use was examined using continuous variables (output expressed per 100 DDDs and 100 days of use), approximate quartiles of dose and duration of PPIs/H_2_RAs use and the combined dose and duration variable. A model detailing total exposure to antisecretory drugs (PPIs and H_2_RAs) was also generated. For presentation purposes the output from the continuous variables was reported per 100 DDDs and 100 days.PPI/H_2_RA use in the 2 years before diagnosis was excluded in all analyses and models were constructed relating to the period since entry into the GPRD and the 5 years before diagnosis/index date.

All analyses were adjusted for potential confounders, including smoking status (unknown, current smoker, non-smoker and ex-smoker), body mass index (unknown, underweight, normal, overweight and obese) according to the World Health Organisation categories (World Health Organisation Working Group, 2009), alcohol use (unknown, current drinker, lifelong non-drinker and ex-drinker), history of pancreatitis, history of diabetes and history of cancer. For smoking, alcohol use and body mass index, the most recent record excluding those within the year prior to the index date was used and for history of diabetes, pancreatitis and cancer we also excluded events within the year prior to the index date. Use of nonsteroidal anti-inflammatory drugs (NSAIDs) or aspirin (ever/never) was accounted for as users are increasingly likely to receive a PPI/H_2_RA for prevention of NSAID-induced ulceration in the GI tract (National Institute for Health and Clinical Excellence, 2008). NSAID/aspirin use has been associated with a reduced risk of pancreatic cancer (Anderson *et al*, 2002). Use of systemic steroids (ever/never use) and hormone replacement therapy (ever/never use) was accounted for, as use of these drugs may confound the association between PPI/H_2_RA and pancreatic cancer. Use of NSAIDs/aspirin, hormone replacement therapy and steroids, in the year before diagnosis, was excluded. Test for trends across categories of dose and duration, where appropriate, was performed. Data on the confounders were not available for all subjects and for those subjects for whom all data were available, a restriction analyses was carried out. All *P*-values were two sided. STATA Version 9 (Timberlake Consultants Ltd, London, UK) was used for all analyses.

## Results

Within the UK GPRD, 1361 pancreatic cancer cases met our initial inclusion criteria and these were matched to 9487 controls. After review of cancer codes, 220 cases (16%) and their controls (1542) were excluded, leaving 1141 pancreatic cancer cases and 7954 matched controls. Each case had at least one matched control and >90% of cases had seven matched controls. The mean duration of UTS follow-up was 10.6 years (s.d., 3.4) for both cases and controls. The mean age at database enrolment was identical for cases and controls at 57.3 years (s.d., 9.8). Over 50% (53.7%) of all study subjects were male (Table 1). The risk of pancreatic cancer was elevated among smokers in our study population OR (95% CI) 1.92 (1.63–2.26) and the OR for pancreatic cancer among subjects with diabetes was 1.90 (95% CI: 1.53–2.35). Any use of a PPI since entry into the GPRD (excluding the 2 years prior to diagnosis) was not associated with the risk of pancreatic cancer, OR (95%CI) 1.02 (0.85–1.22). There was no overall association between pancreatic cancer risk and the total dose of PPI used since entry into the GPRD. The adjusted ORs (95% CI) for an increase in 100 DDDs of PPIs in this period was 0.99 (0.97–1.01). When the dose of PPIs was categorised according to use in controls (approximate quartiles), no association was seen between dose category and pancreatic cancer risk (Table 2). Similar results were observed in the period of 5 years before the index date (excluding the 2 years prior to diagnosis) (data not shown).

Duration of PPI use was examined in a similar manner to dose and no associations with pancreatic cancer risk were seen (Table 3).

Dose and duration of PPI use combined was not associated with pancreatic cancer risk, although there was a suggestion of a reduced risk of pancreatic cancer among those using lower doses of PPIs (Table 4).

No overall association was found between pancreatic cancer risk and the dose and duration of H_2_RAs used since entry into the GPRD (Data not shown). Similar results were seen for use in the 5 years before the index date. The analysis that combined dose and duration of H_2_RA use demonstrated an increased risk of pancreatic cancer, OR (95%CI) 1.25 (1.03–1.52), among short-term users (0–1 years) of high-dose H_2_RAs. However, no increases in risk were seen among those using high-dose H_2_RAs for longer durations of time and the number of these users was small (Table 4).

The total dose and duration of antisecretory drug use combined (PPI plus H_2_RA use) was not associated with pancreatic cancer risk either in the period since entry into the GPRD (Table 5). The results from the restriction analysis completed for all subjects with complete data on confounders showed that the absence of data on some confounders, did not affect the overall estimates (data not shown).

## Discussion

In this large population-based study, no association between risk of pancreatic cancer and antisecretory drug use was seen for ever compared with non-use of PPIs or H_2_RAs. There was also no overall association between pancreatic cancer risk and the total dose or duration of PPIs or H_2_RAs used and no increases in risk were seen among subjects who used these drugs at the highest doses or for the longest durations. No consistent patterns of association were seen when cumulative exposure (dose and duration) to these drugs were examined separately or together (total antisecretory drug use, i.e., PPI plus H_2_RA), although there was some evidence of a decrease in risk among low-dose PPI users and an increase in risk among short-term low-dose H_2_RA users.

Overall, this study does not provide consistent evidence for either an increased or decreased risk of pancreatic cancer associated with the use of PPIs or H_2_RAs. However, few study subjects had long-term exposure to PPIs. Just 16 cases and 122 controls used PPIs (at low or high doses) for longer than 4 years. More profound suppression of acid and subsequent hypergastrinaemia, which occurs with higher doses of PPIs, is biologically more likely to affect pancreatic cancer risk. However, in this study no increased risk of pancreatic cancer was observed among long-term (>4 years) high-dose PPI users, but the number of long-term high-dose PPI users (11 cases, 61 controls) was too small to adequately assess the effect of high doses of these drugs on pancreatic cancer risk.

Certain PPIs were not available in the United Kingdom until 2000 (UK Medicines Information Pharmacist groups, 2001) and the dates of data capture in this study appear to have preceded the rapid increase in use of PPIs in recent years. Pancreatic carcinogenesis has a long latency period (Brat *et al*, 1998), therefore it may be too early to see a relationship between use of PPIs and pancreatic cancer development due to their relatively recent introduction to the UK market.

Since the introduction of PPIs, use of H_2_RAs has declined (National Health Service Business Services Authority, 2009), however, it was deemed possible that some users may have been exposed to H_2_RAs for prolonged periods of time and this may be important for cancer risk. Despite the earlier introduction of H_2_RAs to the UK market (in the mid 1970s), there were very few long-term (>4 years) H_2_RA users (23 cases and 151controls) compared with short-term users (192 cases, 1080 controls) in this study. As PPIs provide a more rapid and sustained increase in gastric pH (Brett, 2005), long-term H_2_RA users may have switched to PPI therapy in recent years, resulting in the reduced number of long-term H_2_RA users.

Epidemiological studies have shown an increased risk of gastric cancer among PPI users (Bateman *et al*, 2003; García Rodríguez *et al*, 2006; Poulsen *et al*, 2009), however, any effect may have been due to reverse causality or confounding by indication. The increasing use of PPIs, in the last decade, was thought to have been related to the sharp rise in oesophageal cancer incidence (Lepage *et al*, 2008; Rosch, 2010). However, increased incidence, along with the higher mortality rates for oesophageal cancer, was attributed to the underlying disease and not to treatment with PPIs (Bateman *et al*, 2003; García Rodríguez *et al*, 2006); Robertson *et al*, 2007). No association between PPI use and colorectal cancer has been observed in large case–control studies (Yang *et al*, 2007; Van soest *et al*, 2008).

Assessment of the relationship between PPI/H_2_RA use and pancreatic cancer risk is likely to be complicated by the underlying indications for use of these drugs. Epidemiological studies have found an increased risk of pancreatic cancer among individuals with duodenal ulcers, gastric ulcers or peptic ulcers (indications for which these drugs are used) (Farrow and Davis, 1990; Luo *et al*, 2007), whereas others have failed to show an association (La Vecchia *et al*, 1990; Silverman *et al*, 1999). *Helicobacter pylori* infection is a leading cause of peptic ulcers and has been shown to be associated with pancreatic cancer risk (Stolzenberg-Solomon *et al*, 2001) as was partial gastrectomy and truncal vagotomy, which are surgical procedures used in the treatment of peptic ulcer disease (Tersmette *et al*, 1990; van Rees *et al*, 1999; Tascilar *et al*, 2002). However, conflicting results have been reported (Caygill *et al*, 1987; La Vecchia *et al*, 1990; Silverman *et al*, 1999).

This investigation has several key strengths. It is the only study to date to assess the association of PPI/H_2_RA exposure and pancreatic cancer. A detailed analysis was possible by stratifying the analyses based on dose, duration, and dose and duration of PPI/ H_2_RA use. The use of prospectively collected prescription data avoids errors of recall and potential recall bias. All subjects in our study had at least 5 years of data available prior to pancreatic cancer diagnosis and data were available prior to diagnosis for a mean of over 10 years. We also adjusted for all major confounders and, although data were not available for all subjects, the results of restriction analyses, including only those patients who had data on these confounders, were not different from the main analyses. The results for the association between pancreatic cancer and smoking and diabetes reported in this study are similar to those obtained in some larger international studies (Heinen *et al*, 2010; Bertuccio *et al*, 2011; Lipworth *et al*, 2011), which supports the validity of our study.

However, the study has some limitations. A low prevalence of long-term PPI/H_2_RA use in the study population meant that we were unable to examine the effects of exposure to these agents for long periods of time. Data on prescriptions issued may not reflect actual use of PPIs/H_2_RAs, but there is no reason to believe that noncompliance with prescription medication would be systematically different between cases and controls. No information was available on over-the-counter PPI/H_2_RA use and misclassification of over-the-counter users as nonusers based on prescription information would have biased the estimates towards the null. However, at the time of this study there was only one PPI-available over-the-counter from pharmacies in the United Kingdom and uptake has been low (Stewart *et al*, 2007), although H_2_RAs are more readily available. Pancreatic cancer diagnoses were not validated in this study and any misclassification of diagnosis is likely to bias the estimate towards the null. However, we excluded very elderly subjects, in whom diagnostic accuracy may be a particular problem, and all cancer codes were reviewed and cases with inconsistent coding were excluded. Furthermore, cancer diagnoses in GPRD appear to be a reliable record of incident cancer diagnoses and have been shown to concord with original medical records in 95% of cases (Jick *et al*, 1997).

In summary, despite biologically plausible mechanisms, whereby use of PPIs or H_2_RAs may increase the risk of pancreatic cancer, we did not observe any consistent associations between use of these drugs and pancreatic cancer risk in a large study involving a representative UK population.

## Figures and Tables

**Table 1:** Characteristics of cases and controls

	**Cases (*n*=1141)**	**Controls (*n*=7954)**	***P***-**value**
Mean age at database enrolment (year) (s.d.)	57.3 (9.8)	57.3 (9.8)	>0.99
Male sex (%)	53.7	53.7	>0.99
Mean duration of follow-up before index date (year) (s.d.)	10.6 (3.4)	10.6 (3.4)	>0.99
			
*Smoking status*			<0.01
Current smoker	302 (26.5%)	1372 (17.3%)	
Non-smoker	463 (40.6%)	3961 (49.8%)	
Ex-smoker	235 (20.6%)	1499 (18.9%)	
Missing data	141 (12.4%)	1122 (14.1%)	
			
*BMI (kg m*^−*2*^)			0.1
<18.5	16 (1.4%)	102 (1.3%)	
⩾18.5 and <25	353 (31.0%)	2323 (29.2%)	
⩾25 and <30	343 (30.1%)	2525 (31.8%)	
⩾30	187 (16.4%)	1108 (13.9%)	
Missing	242 (21.21%)	1896 (23.8%)	
			
*Current drinking status*			0.4
Current drinker	731 (64.1%)	4992 (62.8%)	
Non-drinker	165 (14.5%)	1107 (13.9%)	
Ex-drinker	7 (0.6%)	77 (1.0%)	
Missing data	238 (20.9%)	1778 (22.4%)	
			
*HRT use*			0.3
Never	1095 (95.9%)	7574 (95.2%)	
Ever	46 (4.1%)	380 (4.8%)	
			
*Steroid use*			0.2
Never	990 (86.8%)	6999 (88.0%)	
Ever	151 (13.2%)	955 (12.0%)	
			
*Pancreatitis*			<0.01
Never	1085 (95.1%)	7901 (99.3%)	
Ever	56 (4.9%)	53 (0.7%)	
			
*PPI use*			0.27
Never	964 (84.5%)	6817 (85.7%)	
Ever	177 (15.5%)	1137 (14.3%)	
			
*H*_*2*_ *RA use*			<0.01
Never	877 (76.9%)	6423(80.8%)	
Ever	264 (23.1%)	1531 (19.2%)	
			
*Diabetes*			<0.01
Never	1006 (88.2%)	7442 (93.56%)	
Ever	135 (11.8%)	512 (6.44%)	
			
*Prior cancer*			0.35
Never	1049 (91.9%)	7374 (92.7%)	
Ever	92 (8.1%)	580 (7.3%)	

Abbreviations: BMI=body mass index; H_2_RA=histamine-2-receptor antagonist; HRT=hormone replacement therapy; PPI=proton pump inhibitor.

**Table 2:** Total dose of PPI, H2RA and risk of pancreatic cancer

**PPI use**	**Entry into GPRD until 2 years before index date**				**H_2_RA use**	**Entry into GPRD until 2 years before index date**			
**Dose of PPI use (DDDs)**	**Cases (*n*)**	**Controls (*n*)**	**OR (95% CI)**	**Adjusted OR (95% CI)^a^**	**Dose of H_2_RA use (DDDs)**	**Cases (*n*)**	**Controls (*n*)**	**OR (95% CI)**	**Adjusted OR (95% CI)^a^**
Category 1 (no use)	964	6817	1.0	1.0	Category 1 (no use)	877	6423	1.0	1.0
Category 2 (0–28)	52	311	1.19 (0.88–1.61)	1.14 (0.84–1.55)	Category 2 (0–30)	83	464	1.31 (1.03–1.69)	1.30 (1.01–1.68)
Category 3 (28.5–112)	31	257	0.86 (0.59–1.25)	0.79 (0.54–1.17)	Category 3 (30.5–90)	53	316	1.24 (0.92–1.68)	1.15 (0.84–1.56)
Category 4 (112.5–476)	51	288	1.26 (0.93–1.72)	1.14 (0.83–1.56)	Category 4 (90.5–480)	67	370	1.34 (1.02–1.76)	1.20 (0.91–1.59)
Category 5 (>476)	43	281	1.09 (0.78–1.52)	0.96 (0.68–1.36)	Category 5 (>480)	61	381	1.18 (0.89–1.57)	1.05 (0.78–1.40)

Abbreviations: BMI=body mass index; CI=confidence interval; DDD=defined daily dose; H_2_RA=histamine-2-receptor antagonist; HRT=hormone replacement therapy; NSAID=nonsteroidal anti-inflammatory drug; OR=odds ratio; PPI=proton pump inhibitor.

a Adjusted for smoking status (current smoker, non-smoker, ex-smoker, missing data), BMI (<18.5, ⩾18.5, <25, ⩾25, <30 and ⩾30 kg m^−2^, missing), alcohol use (current drinker, non-drinker, ex-drinker), history of chronic pancreatitis (ever/never), use of other drugs (NSAIDs, steroids and HRT (ever/never)), diabetes (ever/never) and prior cancer (ever/never).

**Table 3:** Duration of PPI, H_2_RA use and risk of pancreatic cancer

**PPI use**	**Entry into GPRD until 2 years before index date**				**H_2_RA use**	**Entry into GPRD until 2 years before index date**			
**Duration of PPI use (days)**	**Cases (*n*)**	**Controls (*n*)**	**OR (95% CI)**	**Adjusted OR (95%CI)^a^**	**Duration of H_2_RA use (Days)**	**Cases (*n*)**	**Controls (*n*)**	**OR (95% CI)**	**Adjusted OR (95% CI)^a^**
-Category 1 (no use)	964	6817	1.0	1.0	-Category 1 (no use)	877	6424	1.0	1.0
-Category 2 (0–28)	51	308	1.18 (0.87–1.60)	1.13 (0.83–1.55)	-Category 2 (0–30)	80	449	1.31 (1.02–1.69)	1.30 (1.01–1.69)
-Category 3 (28.5–106)	37	264	1.0 (0.70–1.42)	0.92 (0.64–1.32)	-Category 3 (30.5–90)	57	320	1.32 (0.98–1.77)	1.20 (0.89–1.63)
-Category 4 (106.5–552)	47	281	1.20 (0.87–1.65)	1.07 (0.77–1.48)	-Category 4 (90.5–520)	71	379	1.39 (1.06–1.81)	1.26 (0.96–1.65)
-Category 5 (>552)	42	284	1.05 (0.75–1.47)	0.93 (0.65–1.32)	-Category 5 (>520)	56	382	1.08 (0.81–1.44)	0.95 (0.71–1.29)

Abbreviations: BMI=body mass index; CI=confidence interval; DDD=defined daily dose; H_2_RA=Histamine-2-receptor antagonist; HRT=hormone replacement therapy; NSAID=nonsteroidal anti-inflammatory drug; OR=odds ratio; PPI=proton pump inhibitor.

a Adjusted for smoking status (current smoker, non-smoker, ex-smoker, missing data), BMI (<18.5, ⩾18.5, <25, ⩾25, <30 and ⩾30 kg m^−2^ , missing), alcohol use (current drinker, non-drinker, ex-drinker), history of chronic pancreatitis (ever/never), use of other drugs (NSAIDs, steroids and HRT (ever/never)), diabetes (ever/never) and prior cancer (ever/never).

**Table 4:** Pancreatic cancer risk according to dose and duration of PPI, H_2_RA use since entry into GP

	**<1.0 DDD per day**				**⩾1.0 DDD per day**			
	**Cases *n***	**Controls *n***	**OR (95% CI)**	**Adjusted OR (95%CI)^a^**	**Cases *n***	**Controls *n***	**OR (95% CI)**	**Adjusted OR (95%CI)^a^**
*(a) PPI*								
*Duration of PPI use*								
Nonusers	964	6817	1.0	1.0	964	6817	1.0	1.0
0–1 year	20	195	0.70 (0.44–1.12)	0.60 (0.37–0.98)	107	592	1.27 (1.02–1.58)	1.20 (0.95–1.50)
1–4 years	14	113	0.87 (0.50–1.54)	0.70 (0.39–1.27)	20	115	1.23 (0.76–1.99)	1.08 (0.65–1.77)
>4 years	5	61	0.61 (0.24–1.53)	0.54 (0.20–1.42)	11	61	1.32 (0.69–2.55)	1.32 (0.67–2.60)
								
*(b) H*_*2*_ *RA*								
*Duration of H*_*2*_*RA use*								
Nonusers	877	6424	1.0	1.0	877	6424	1.0	1.0
0–1 years	29	193	1.09 (0.72–1.63)	0.98 (0.65–1.49)	163	887	1.34 (1.12–1.61)	1.25 (1.03–1.52)
1–4 years	13	109	0.91 (0.5–1.63)	0.92 (0.51–1.68)	36	190	1.40 (0.97–2.01)	1.24 (0.84–1.81)
>4 years	9	80	0.96 (0.47–1.96)	0.82 (0.39–1.72)	14	71	1.54 (0.86–2.77)	1.35 (0.73–2.48)

Abbreviations: BMI=body mass index; CI=confidence interval; DDD=defined daily dose; H_2_RA=histamine-2-receptor antagonist; HRT=hormone replacement therapy; NSAID=nonsteroidal anti-inflammatory drug; OR=odds ratio; PPI=proton pump inhibitor.

a Adjusted for smoking status (current smoker, non-smoker, ex-smoker, missing data), BMI (<18.5, ⩾18.5, <25, ⩾25, <30 and ⩾30 kg m^−2^ , missing), alcohol use (current drinker, non-drinker, ex-drinker), history of chronic pancreatitis (ever/never), use of other drugs (NSAIDs, steroids and HRT (ever/never)), diabetes (ever/never) and prior cancer (ever/never).

**Table 5:** Pancreatic risk according to total dose and duration of acidsuppressive therapy (PPIs and H_2_RAs) since entry into the GPRD

**PPIs and H_2_Ras**	**Entry into GPRD until 2 years before index date**			**PPIs and H2RAs**	**Entry into GPRD until 2 years before index date**		
**Dose (DDDs)**	**Cases**	**Controls**	**Adjusted OR (95% CI)^a^**	**Duration (Days)**	**Cases**	**Controls**	**Adjusted OR (95% CI)^a^**
No use	818	5966	1.0	-No use	818	5968	10
Category 2 (0–30)	77	500	1.12 (0.86–1.45)	-Category 2 (0–37)	78	502	1.15 (0.89–1.49)
Category 3 (31–144)	81	496	1.12 (0.87–1.44)	-Category 3 (37–144)	84	492	1.14 (0.89–1.47)
Category 4 (145–776)	89	495	1.21 (0.95–1.55)	-Category 4 (145–849)	84	496	1.16 (0.90–1.49)
Category 5 (>776)	76	497	0.97 (0.74–1.27)	-Category 5 (>849)	77	496	1.03 (0.79–1.35)

Abbreviations: BMI=body mass index; CI=confidence interval; DDD=defined daily dose; H_2_RA=Histamine-2-receptor antagonist; HRT=hormone replacement therapy; NSAID=nonsteroidal anti-inflammatory drug; OR=odds ratio; PPI=proton pump inhibitor.

a Adjusted for smoking status (current smoker, non-smoker, ex-smoker, missing data), BMI (<18.5, ⩾18.5, <25, ⩾25, <30 and ⩾30 kg m^−2^, missing), alcohol use (current drinker, non-drinker, ex-drinker), history of chronic pancreatitis (ever/never), use of other drugs (NSAIDs, steroids and HRT (ever/never)), diabetes (ever/never) and prior cancer (ever/never).

## References

[bib1] Anderson KE, Johnson TW, Lazovich D, Folsom AR (2002) Association between nonsteroidal anti-inflammatory drug use and the incidence of pancreatic cancer. J Natl Cancer Inst 94: 1168–1171 12165642 10.1093/jnci/94.15.1168

[bib2] Bateman DN, Colin-Jones D, Hartz S, Langman M, Logan RF, Mant J, Murphy M, Paterson KR, Rowsell R, Thomas S, Vessey M (2003) Mortality study of 18 000 patients treated with omeprazole. Gut 52: 942–946 12801948 10.1136/gut.52.7.942 PMC1773704

[bib3] Bertuccio P, La Vecchia C, Silverman DT, Petersen GM, Bracci PM, Negri E, Li D, Risch HA, Olson SH, Gallinger S, Miller AB, Bueno-de-Mesquita HB, Talamini R, Polesel J, Ghadirian P, Baghurst PA, Zatonski W, Fontham ET, Bamlet WR, Holly EA, Lucenteforte E, Hassan M, Yu H, Kurtz RC, Cotterchio M, Su J, Maisonneuve P, Duell EJ, Bosetti C, Boffetta P (2011) Cigar and pipe smoking, smokeless tobacco use and pancreatic cancer: an analysis from the International Pancreatic Cancer Case-Control Consortium (PanC4). Ann Oncol 22(6): 1420–1426 21245160 10.1093/annonc/mdq613 PMC3139985

[bib4] BMJ group (2009) Proton pump inhibitors. In British National Formulary, Section 1.3.5, pp 48–50. RPS publishing, GGP Media: Germany

[bib5] Brat DJ, Lillemoe KD, Yeo CJ, Warfield PB, Hruban RH (1998) Progression of pancreatic intraductal neoplasias to infiltrating adenocarcinoma of the pancreas. Am J Surg Pathol 22: 163–169 9500216 10.1097/00000478-199802000-00003

[bib6] Brett S (2005) Science review: the use of proton pump inhibitors for gastric acid suppression in critical illness. Crit Care 9: 45–50 15693983 10.1186/cc2980 PMC1065099

[bib7] Caygill CP, Hill MJ, Hall CN, Kirkham JS, Northfield TC (1987) Increased risk of cancer at multiple sites after gastric surgery for peptic ulcer. Gut 28: 924–928 3666558 10.1136/gut.28.8.924 PMC1433152

[bib8] Chao C, Hellmich MR (2010) Gastrin, inflammation, and carcinogenesis. Curr Opin Endocrinol Diabetes Obes 17: 33–39 19907321 10.1097/MED.0b013e328333faf8 PMC3970765

[bib9] Chau I, Cunningham D, Russell C, Norman AR, Kurzawinski T, Harper P, Harrison P, Middleton G, Daniels F, Hickish T, Prendeville J, Ross PJ, Theis B, Hull R, Walker M, Shankley N, Kalindjian B, Murray G, Gillbanks A, Black J (2006) Gastrazole (JB95008), a novel CCK2/gastrin receptor antagonist, in the treatment of advanced pancreatic cancer: results from two randomised controlled trials. Br J Cancer 94: 1107–1115 16622436 10.1038/sj.bjc.6603058 PMC2361246

[bib10] Chu M, Kullman E, Rehfeld JF, Borch K (1997) Effect of chronic endogenous hypergastrinaemia on pancreatic growth and carcinogenesis in the hamster. Gut 40: 536–540 9176085 10.1136/gut.40.4.536 PMC1089284

[bib11] DeVault KR, Castell DO (2005) Updated guidelines for the diagnosis and treatment of gastroesophageal reflux disease. Am J Gastroenterol 100: 190–200 15654800 10.1111/j.1572-0241.2005.41217.x

[bib12] Farrow DC, Davis S (1990) Risk of pancreatic cancer in relation to medical history and the use of tobacco, alcohol and coffee. Int J Cancer 45: 816–820 2335385 10.1002/ijc.2910450504

[bib13] Fried M, Siegrist H, Frei R, Froehlich F, Duroux P, Thorens J, Blum A, Bille J, Gonvers JJ, Gyr K (1994) Duodenal bacterial overgrowth during treatment in outpatients with omeprazole. Gut 35: 23–26 8307444 10.1136/gut.35.1.23 PMC1374626

[bib14] García Rodríguez LA, Pérez Gutthann S (1998) Use of the UK General Practice Research Database for pharmacoepidemiology. Br J Clin Pharmacol 45: 419–425 9643612 10.1046/j.1365-2125.1998.00701.x PMC1873548

[bib15] García Rodríguez LA, Lagergren J, Lindblad M (2006) Gastric acid suppression and risk of oesophageal and gastric adenocarcinoma: a nested case control study in the UK. Gut 55: 1538–1544 16785284 10.1136/gut.2005.086579 PMC1860118

[bib16] Heinen MM, Verhage BA, Goldbohm RA, van den Brandt PA (2010) Active and passive smoking and the risk of pancreatic cancer in the Netherlands Cohort Study. Cancer Epidemiol Biomarkers Prev 19(6):: 1612–1622 20501775 10.1158/1055-9965.EPI-10-0121

[bib17] Howatson AG, Carter DC (1987) Pancreatic carcinogenesis: effect of secretin in the hamster-nitrosamine model. J Natl Cancer Inst 78: 101–105 3467121 10.1093/jnci/78.1.101

[bib18] IMS Health (2008) Top Therapeutic Classes by US Sales. Available from: http://www.imshealth.com/deployedfiles/imshealth/Global/Content/StaticFile/Top_Line_Data/2008_Top_Therapy_Classes_by_U.S._Sales.pdf (accessed 7 September 2009)

[bib19] Jemal A, Siegel R, Ward E, Murray T, Xu J, Thun MJ (2007) Cancer statistics 2007. CA Cancer J Clin 57: 43–66 17237035 10.3322/canjclin.57.1.43

[bib20] Jick H, Jick SS, Derby LE (1991) Validation of information recorded on general practicioner based computersied data resource in the United Kingdom. BMJ 302: 766–768 2021768 10.1136/bmj.302.6779.766 PMC1669537

[bib21] Jick H, Jick S, Derby LE, Vasilakis C, Myers MW, Meier CR (1997) Calcium channel blockers and risk of cancer. Lancet 349: 525–528 9048789 10.1016/S0140-6736(97)80084-0

[bib22] Jick H, Terris BZ, Derby LE, Jick SS (1992) Further validation of information recorded on a general practicioner based computerised data resource in the United Kingdom. Pharmacoepidemiol Drug Saf 1: 347–349

[bib23] Kinnear H, Gavin A, Mole D, Ranaghan L (2005) Cancer Services Audit 2001, Pancreas. N Ireland Cancer Registry

[bib24] Klinkenberg-Knol EC, Festen HP, Jansen JB, Lamers CB, Nelis F, Snel P, Lückers A, Dekkers CP, Havu N, Meuwissen SG (1994) Long-term treatment with omeprazole for refractory reflux esophagitis: efficacy and safety. Ann Intern Med 121: 161–167 8017742 10.7326/0003-4819-121-3-199408010-00001

[bib25] La Vecchia C, Negri E, D'Avanzo B, Ferraroni M, Gramenzi A, Savoldelli R, Boyle P, Franceschi S (1990) Medical history, diet and pancreatic cancer. Oncology 47: 463–466 2243664 10.1159/000226872

[bib26] Lepage C, Rachet B, Jooste V, Faivre J, Coleman MP (2008) Continuing rapid increase in esophageal adenocarcinoma in England and Wales. Am J Gastroenterol 103: 2694–2699 18853967 10.1111/j.1572-0241.2008.02191.x

[bib27] Lipworth L, Zucchetto A, Bosetti C, Franceschi S, Talamini R, Serraino D, McLaughlin JK, La Vecchia C, Negri E (2011) Diabetes mellitus, other medical conditions and pancreatic cancer: a case-control study. Diabetes Metab Res Rev 27(3):: 255–261 21309046 10.1002/dmrr.1162

[bib28] Luo J, Nordenvall C, Nyrén O, Adami HO, Permert J, Ye W (2007) The risk of pancreatic cancer in patients with gastric or duodenal ulcer disease. Int J Cancer 120: 368–372 17044024 10.1002/ijc.22123

[bib29] National Health Service Business Services Authority (2009) Update on growth of prescription volume and cost in the year to March 2009. Available from: http://www.nhsbsa.nhs.uk/PrescriptionServices/Documents/Volume_and_cost_year_to_March_2009.pdf (accessed 21 August 2009)

[bib30] National Institute for Health and Clinical Excellence (2008) The care and management of osteoarthritis in adults. Available at: http://www.nice.org.uk/nicemedia/pdf/CG59NICEguideline.pdf (accessed 21 July 2009)

[bib31] Naunton M, Peterson GM, Bleasel MD (2000) Overuse of proton pump inhibitors. J Clin Pharm Ther 25: 333–340 11123484 10.1046/j.1365-2710.2000.00312.x

[bib32] Parsa I (1987) Invitro carcinogenesis of the pancreas. In Experimental pancreatic cacrcinogenesis, Scarpell, D.G, Reddy, J.K and Longnecker, D.S (eds) CRC press Boca Raton: Florida. 209–232

[bib33] Parsa I, Marsh WH, Sutton AL (1981) An *in vitro* model of human pancreas carcinogenesis: effects of nitroso compounds. Cancer 47: 1543–1551 6168353 10.1002/1097-0142(19810315)47:6+<1543::aid-cncr2820471417>3.0.co;2-3

[bib34] Poulsen AH, Christensen S, McLaughlin JK, Thomsen RW, Sørensen HT, Olsen JH, Friis S (2009) Proton pump inhibitors and risk of gastric cancer: a population-based cohort study. Br J Cancer 100: 1503–1507 19352380 10.1038/sj.bjc.6605024 PMC2694435

[bib35] Risch HA (2003) Etiology of pancreatic cancer, with a hypothesis concerning the role of N-nitroso compounds and excess gastric acidity. J Natl Cancer Inst 95: 948–960 12837831 10.1093/jnci/95.13.948

[bib36] Roberts S, Griffein EP, Harper EA, Hull RAD, Kalindjian SB, Lilley EJ, Kotecha A, Shankley NP, Sykes DA, Watt GF, Black JW (2002) JB95008 (gastrazole), a selective CCK_2_ receptor (CCK_2_R) antagonist with *in vivo* activity. Pharmacologist 44, abstract no. 30.4

[bib37] Robertson DJ, Larsson H, Friis S, Pedersen L, Baron JA, Sørensen HT (2007) Proton pump inhibitor use and risk of colorectal cancer: a population-based, case-control study. Gastroenterology 133: 755–760 17678921 10.1053/j.gastro.2007.06.014

[bib38] Rosch PJ (2010) Could proton pump inhibitors cause cancer? Arch Intern Med 170: 1775–1776 20975029 10.1001/archinternmed.2010.375

[bib39] Sarfati PD, Genik P, Morisset J (1985) Caerulein and secretin induced pancreatic growth: a possible control by endogenous pancreatic somatostatin. Regul Pept 11: 261–273 2865774 10.1016/0167-0115(85)90058-8

[bib40] Silverman DT, Schiffman M, Everhart J, Goldstein A, Lillemoe KD, Swanson GM, Schwartz AG, Brown LM, Greenberg RS, Schoenberg JB, Pottern LM, Hoover RN, Fraumeni Jr JF (1999) Diabetes mellitus, other medical conditions and familial history of cancer as risk factors for pancreatic cancer. Br J Cancer 80: 1830–1837 10468306 10.1038/sj.bjc.6690607 PMC2363127

[bib41] Smith JP, Fantaskey AP, Liu G, Zagon IS (1995) Identification of gastrin as a growth peptide in human pancreatic cancer. Am J Physiol 268: 135–141 10.1152/ajpregu.1995.268.1.R135 7840313

[bib42] Smith JP, Liu G, Soundararajan V, McLaughlin PJ, Zagon IS (1994) Identification and characterization of CCK-B/gastrin receptors in human pancreatic cancer cell lines. Am J Physiol 266: 277–283 10.1152/ajpregu.1994.266.1.R277 8304551

[bib43] Stewart D, John D, Cunningham S, McCaig D, Hansford D (2007) A comparison of community pharmacists′ views of over-the-counter omeprazole and simvastatin. Pharmacoepidemiol Drug Saf 16: 1290–1297 17918161 10.1002/pds.1481

[bib44] Stolzenberg-Solomon RZ, Blaser MJ, Limburg PJ, Perez-Perez G, Taylor PR, Virtamo J, Albanes D (2001) Helicobacter pylori seropositivity as a risk factor for pancreatic cancer. J Natl Cancer Inst 93: 937–941 11416115 10.1093/jnci/93.12.937

[bib45] Tascilar M, van Rees BP, Sturm PD, Tytgat GN, Hruban RH, Goodman SN, Giardiello FM, Offerhaus GJ, Tersmette AC (2002) Pancreatic cancer after remote peptic ulcer surgery. J Clin Pathol 55: 340–345 11986336 10.1136/jcp.55.5.340 PMC1769656

[bib46] Tersmette AC, Offerhaus GJ, Giardiello FM, Tersmette KW, Vandenbroucke JP, Tytgat GN (1990) Occurrence of non-gastric cancer in the digestive tract after remote partial gastrectomy: analysis of an Amsterdam cohort. Int J Cancer 46: 792–795 2228307 10.1002/ijc.2910460507

[bib47] Thorens J, Froehlich F, Schwizer W, Saraga E, Bille J, Gyr K, Duroux P, Nicolet M, Pignatelli B, Blum AL, Gonvers JJ, Fried M (1996) Bacterial overgrowth during treatment with omeprazole compared with cimetidine: a prospective randomised double blind study. Gut 39: 54–59 8881809 10.1136/gut.39.1.54 PMC1383231

[bib48] UK Medicines Information Pharmacist groups (2001) New medicines on the market. Available at: http://www.ukmi.nhs.uk/NewMaterial/html/docs/esomep.pdf (accessed 9 August 2009)

[bib49] van Rees BP, Tascilar M, Hruban RH, Giardiello FM, Tersmette AC, Offerhaus GJ (1999) Remote partial gastrectomy as a risk factor for pancreatic cancer: potential for preventive strategies. Ann Oncol 10(Suppl 4): 204–207 10436823

[bib50] van Soest EM, van Rossum LG, Dieleman JP, van Oijen MG, Siersema PD, Sturkenboom MC, Kuipers EJ (2008) Proton pump inhibitors and the risk of colorectal cancer. Am J Gastroenterol 103: 966–973 18070237 10.1111/j.1572-0241.2007.01665.x

[bib51] Vermeer IT, Engels LG, Pachen DM, Dallinga JW, Kleinjans JC, van Maanen JM (2001) Intragastric volatile N-nitrosamines, nitrite, pH, and Helicobacter pylori during long-term treatment with omeprazole. Gastroenterology 121: 517–525 11522734 10.1053/gast.2001.27098

[bib52] World Health Organisation Working Group (2008) About the ATC/DDD system, 2009. Available at: http://www.whocc.no/atcddd/atcsystem.html (accessed 22 July 2009)

[bib53] World Health Organisation Working Group (2009) Global Database on Body Mass Index: BMI Classification: WHO 2003. Available at: http://www.who.int/bmi/index.jsp?introPage=intro_3.html (accessed 14 July 2009)

[bib54] Wynn GH, Sandson NB, Cozza KL (2007) Gastrointestinal medications. Psychosomatics 48: 79–85 17209156 10.1176/appi.psy.48.1.79

[bib55] Yang YX, Hennessy S, Propert K, Hwang WT, Sedarat A, Lewis JD (2007) Chronic proton pump inhibitor therapy and the risk of colorectal cancer. Gastroenterology 133: 748–754 17678926 10.1053/j.gastro.2007.06.022

[bib56] Yeomans ND, Brimblecombe RW, Elder J, Heatley RV, Misiewicz JJ, Northfield TC, Pottage A (1995) Effects of acid suppression on microbial flora of upper gut. Dig Dis Sci 40: 81S–95S 7859586 10.1007/BF02214873

